# Gut virome orchestrates nitrogen partitioning between the host and microbiome

**DOI:** 10.1002/imt2.70177

**Published:** 2026-10-04

**Authors:** Shujie Xu, Xianglin Fei, Fengqing Lin, Wenzi Wu, Jiachen Zhuang, Qiufen Mo, Xiuan Zhan, Aikun Fu

**Affiliations:** ^1^ Zhejiang Key Laboratory of nutrition and breeding for high‐quality animal products, College of Animal Sciences Zhejiang University Hangzhou China; ^2^ Department of Critical Care Medicine, Jinling Hospital, Affiliated Hospital of Medical School Nanjing University Nanjing China; ^3^ National Key Laboratory for Development and Utilization of Forest Food Resources, College of Food and Health Zhejiang A&F University Hangzhou China

**Keywords:** gut virome, nitrogen metabolism, phage‐bacteria interaction, protein restriction

## Abstract

Dietary protein is essential for host growth and nitrogen homeostasis. How dietary protein shapes intestinal phage–bacteria interactions and influences the translation of dietary cues into host physiological outcomes remains poorly understood. We show that low‐protein diet (LPD) substantially remodels the murine gut virome by altering phage–bacteria dynamics. Under LPD, nutritional stress suppresses bacterial phage release without altering the predicted proportion of lysogenic phages, leading to reduced free phage abundance and weaker bacterial lysis. Lysogenic phages carrying carbohydrate‐related auxiliary metabolic genes (AMGs) may promote expansion of the bacterial host population while suppressing urease‐mediated nitrogen recycling. High bacterial load coupled with low free phage availability creates a nitrogen trap that both sequesters host‐accessible nitrogen and accelerates urea loss, thereby constraining host growth. Both the normal diet (ND) virome, defined by its high free phage abundance and lytic potential, and an equivalently sized LPD phage pool partially rescued nitrogen flow in mice fed a LPD. These results reveal a previously unrecognized gut “viral shunt,” in which balanced phage–bacteria interactions redistribute nitrogen within the gut. Together, this study highlights the ecological significance of phage–bacteria interactions in host nutritional adaptation and suggests that targeted viral manipulation may offer a potential therapeutic strategy for protein‐deficiency‐related disorders.

## Highlights

- Under normal protein intake, lytic phages promote bacterial lysis and release microbial nitrogen that can be assimilated by the host.
- Protein restriction favors a lysogenic phage strategy, reducing bacterial lysis and microbial protein release and thereby limiting nitrogen availability to the host.
- Protein restriction enriches phage‐carried carbohydrate metabolism‐associated auxiliary metabolic genes, reshaping bacterial metabolism and impairing intestinal urea nitrogen recycling.

## Graphical abstract

Under normal protein intake, lytic phages promote bacterial turnover, releasing microbial nitrogen that can be assimilated by the host and thereby revealing phage‐mediated lysis as a potential pathway for host nutrient acquisition. Under protein restriction, however, phages shift toward a lysogenic strategy, reducing bacterial death and microbial protein release and ultimately limiting nitrogen availability to the host. Concurrently, protein restriction promotes the expansion of carbohydrate‐utilizing bacteria in association with phages carrying carbohydrate metabolism‐associated auxiliary metabolic genes, which further constrains ureolytic bacterial niches and thereby impairs intestinal urea nitrogen recycling.

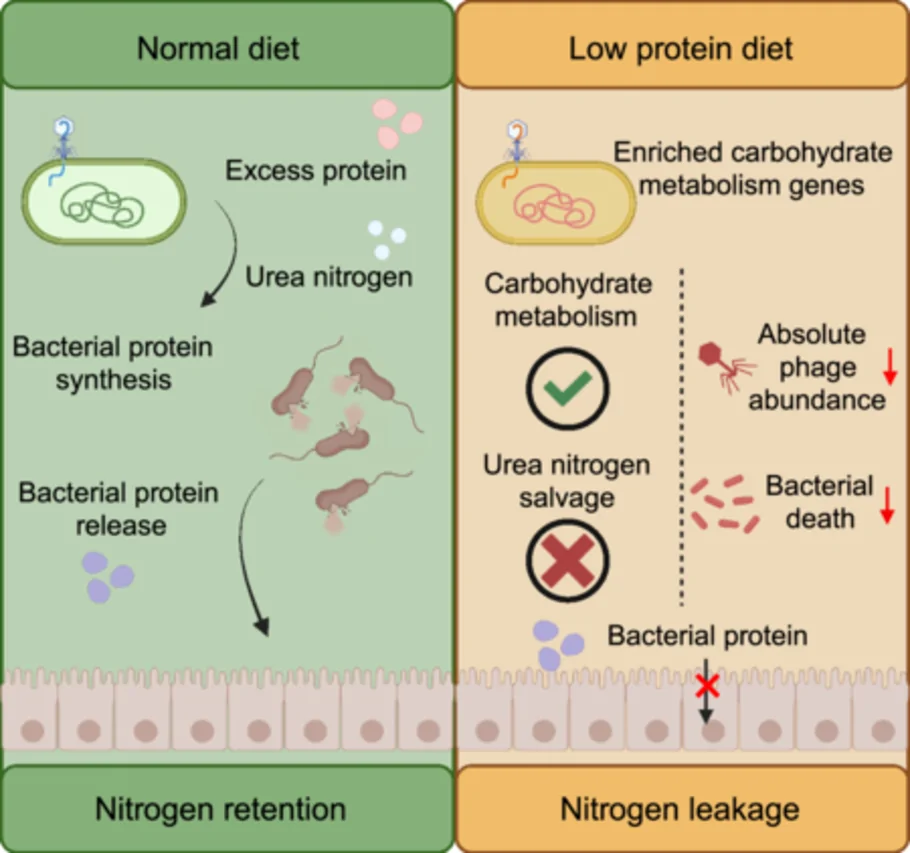

## INTRODUCTION

Protein–energy malnutrition (PEM) during childhood is associated with impaired physical growth and developmental outcomes [1, 2]. Diets associated with PEM are often dominated by carbohydrates and severely limited in protein, creating a chronic imbalance between energy and nitrogen availability [3]. Host–microbe interactions are central to nutrient metabolism, and metagenomic studies suggest that microbial dysbiosis in malnourished children may actively drive nutrient deficiency and persistent growth failure [4]. Under physiological conditions, mammals rely on a symbiotic process known as urea nitrogen salvage (UNS) to maintain nitrogen homeostasis [5]. In this canonical mutualistic pathway, host‐derived urea is secreted into the intestinal lumen, where urease‐expressing gut bacteria hydrolyze urea into ammonia that can be reabsorbed and incorporated into non‐essential amino acids [6]. This mechanism provides a critical buffer against fluctuations in dietary protein intake. However, evidence indicates that under low‐protein diet (LPD) conditions, bacterial urease activity is markedly reduced [7], compromising UNS efficiency and potentially limiting the host's capacity to recycle endogenous nitrogen. The failure of this compensatory pathway has been closely linked to impaired growth and may contribute to irreversible developmental deficits.

The collapse of UNS in malnutrition reflects a deeper breakdown of host–microbe metabolic cooperation. Extensive metagenomic analyses have documented shifts in bacterial community composition and functional potential in undernourished hosts [8, 9, 10], yet the upstream drivers of these changes remain poorly understood. One largely unexplored candidate is the gut virome, which is dominated by phages and increasingly recognized as a key regulator of microbial ecosystems [11]. By infecting and regulating bacterial populations, phages are increasingly recognized not merely as passive members of the microbiota but as key determinants of bacterial community assembly and metabolic function [12, 13]. Phages can influence bacterial metabolism through two non‐mutually exclusive mechanisms, either by encoding auxiliary metabolic genes (AMGs) that reprogram host metabolic pathways [14], or by selectively lysing dominant bacterial populations under “kill‐the‐winner” dynamics [15], reshaping nutrient flow at the community level. Given that bacterial urease activity and nitrogen recycling are largely determined by the abundance, composition, and physiological state of ureolytic bacteria, phage‐mediated regulation of bacterial populations may represent an additional mechanism influencing nitrogen availability within the gut ecosystem [16]. Moreover, recent studies have shown that dietary factors can reshape gut virome composition, suggesting that changes in nutrient availability, including dietary protein levels, may alter phage–bacterium interactions and microbial nutrient utilization [17, 18]. Despite these theoretical frameworks, whether and how the gut virome contributes to metabolic dysfunction under LPD conditions has not been systematically investigated.

Using multi‐omics, fecal virome transplantation (FVT), and ^15^N tracing, the present study reveals how the gut virome regulates host nitrogen metabolism under protein restriction. LPD alters the gut virome, with enrichment of phage‐encoded AMGs associated with increased bacterial carbohydrate utilization and reduced urease‐dependent nitrogen salvage. Virome transplantation from normally fed donors rescues host growth despite persistent UNS impairment. Collectively, elevated phage predation triggers a “gut viral shunt,” releasing bacterial proteins as an alternative nitrogen source. These findings establish the phageome as an ecological buffer, compensating for classical enzymatic mutualism failure under nitrogen‐limited conditions.

## RESULT

### LPD alters the gut virome and impairs UNS

To systematically investigate how host nutritional status modulates the “phage–bacteria” interaction network, we established a LPD mouse model. Dietary crude protein was reduced from 19.4% in the normal diet (ND) to 6% in the LPD, with carbohydrates used to compensate for the reduced protein content, while maintaining constant proportions of other nutrients (Figure 1A). Compared with mice in the ND group, 21‐day‐old weaned mice exposed to LPD exhibited pronounced growth retardation as the markedly restricted body weight gain, body length, and femur length were reduced, and feed‐to‐gain ratio (F/G) increased markedly (*p* < 0.05, Figure 1B–E), indicating severe impairment of host anabolic capacity and nutrient utilization efficiency.

**FIGURE 1:**
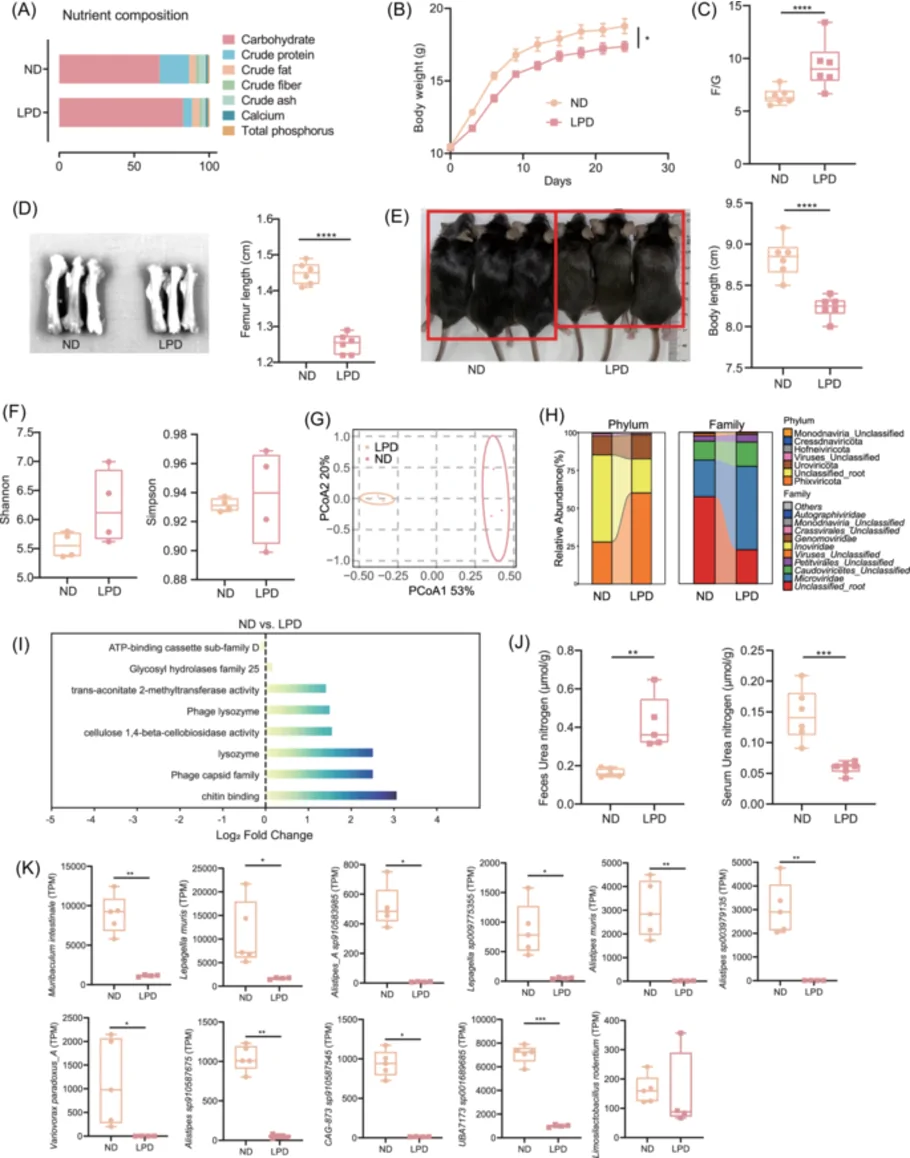
LPD impairs growth performance and reshapes the gut virome. (A) Nutrient composition of normal diet (ND) and low‐protein diet (LPD). Physiological effects of LPD on weaned mice: (B) body weight changes; (C) F/G; (D) femur images with length quantification; (E) dorsal images of mice with body length measurement (*n* = 6). Gut virome analysis: (F) alpha diversity (Shannon and Simpson indices); (G) Principal Coordinate Analysis (PCoA) based on Bray–Curtis distances; (H) viral taxonomic composition at the phylum and family levels (*n* = 4).(I) Differential abundance of viral auxiliary metabolic genes (AMGs) between ND and LPD (log2 fold change), with enrichment in genes related to carbohydrate metabolism in LPD (*n* = 4). (J) Serum and fecal urea nitrogen concentrations, indicating impaired nitrogen recycling in LPD mice (*n* = 6). (K) Abundance of bacteria harboring complete urease gene clusters in the caeca of ND and LPD mice (*n* = 4 or 5). For panels B, data are presented as mean ± SEM. For panels C, D, E, F, J and K, data are shown as box plots. Statistical significance was assessed using two‐tailed Student's *t*‐test or Welch's *t*‐test where appropriate; non‐parametric comparisons were performed using the Mann–Whitney *U* test (*p < 0.05, **p < 0.01, ***p < 0.001, ****p < 0.0001).

To explore the microbial basis of these phenotypes, cecal contents were analyzed using virus‐like particle‐enriched metagenomic sequencing. Virome sequencing revealed that LPD did not significantly alter virome α‐diversity (Shannon and Simpson indices, *p* > 0.05; Figure 1F). β‐diversity analysis based on Bray–Curtis distances confirmed a significant separation between the ND and LPD gut virome (Figure 1G), indicating diet‐specific remodeling of gut virome composition. Taxonomically, LPD induced a marked expansion of *Microviridae* at the family level (Figure 1H). Functional annotation of viral genomes (COG) revealed that putative AMGs were significantly enriched for carbohydrate metabolism functions in the LPD virome (Figure 1I).

Although bacterial α‐diversity remained largely unchanged, PCoA demonstrated that LPD significantly reshaped bacterial community structure (Figure S1A,B). Under LPD, bacteria capable of utilizing host‐derived glycoproteins or non‐protein substrates were selectively enriched, including *Akkermansia* and *Acetatifactor*. Specifically, *A. muciniphila* utilizes host mucins as carbon and nitrogen sources and *Acetatifactor* is enriched in glycoside hydrolase genes (Figure S1C) [19, 20, 21]. Concomitantly, functional genomic analysis of the gut microbiome revealed a global metabolic shift under LPD conditions. Genes involved in carbohydrate metabolism, including glycosyl transferases (GT) and glycoside hydrolases (GHs), were markedly enriched in the LPD microbiome (Figure S1D). By contrast, this shift came at the expense of nitrogen cycling capacity, as key modules related to urease activity and nitrogen metabolism were systematically downregulated under LPD conditions (Figure S1E). Notably, functions associated with urea degradation were markedly reduced, including urease metallocenter assembly, urease subunit proteins (alpha, beta, and gamma), and the assembly of the nickel‐dependent urease active site (Figure S1E).

These metagenomic findings were mirrored by host metabolic phenotypes, as LPD‐fed mice exhibited a paradoxical pattern characterized by increased fecal urea nitrogen and reduced serum urea nitrogen (Figure 1J). The rise in fecal urea reflects impaired microbial urease activity, limiting the hydrolysis and recycling of host‐derived urea, whereas the decrease in serum urea results from insufficient amino acid availability and reduced hepatic urea cycle activity. We hypothesized that expansion of carbohydrate‐utilizing bacteria competitively excluded urease‐producing taxa via niche overlap. Accordingly, we quantified urea‐degrading bacteria defined by complete urease gene clusters and found that, except for *Limosilactobacillus rodentium*, their abundance was significantly higher in the ND group than in the LPD group (*p* < 0.05, Figure 1K). Overall, LPD remodels the “phage–bacteria” network and systematically disrupts intestinal microbial nitrogen recycling, thereby exacerbating host nitrogen loss and growth restriction under protein deficiency.

### LPD‐derived virome alters bacterial metabolism and impairs UNS to drive host growth retardation

To investigate the contribution of the virome to the observed metabolic phenotype and assess whether it influences host growth through bacterial community regulation, we performed FVT experiments (Figure 2A). Transplantation of LPD‐derived virome into ND mice (FVT‐LPD, FL group) reproduced key features of the donor's growth‐impaired phenotype, whereas ND mice receiving ND‐derived virome (ND + ND) showed no significant change in body weight (Figure 2B–E).

**FIGURE 2:**
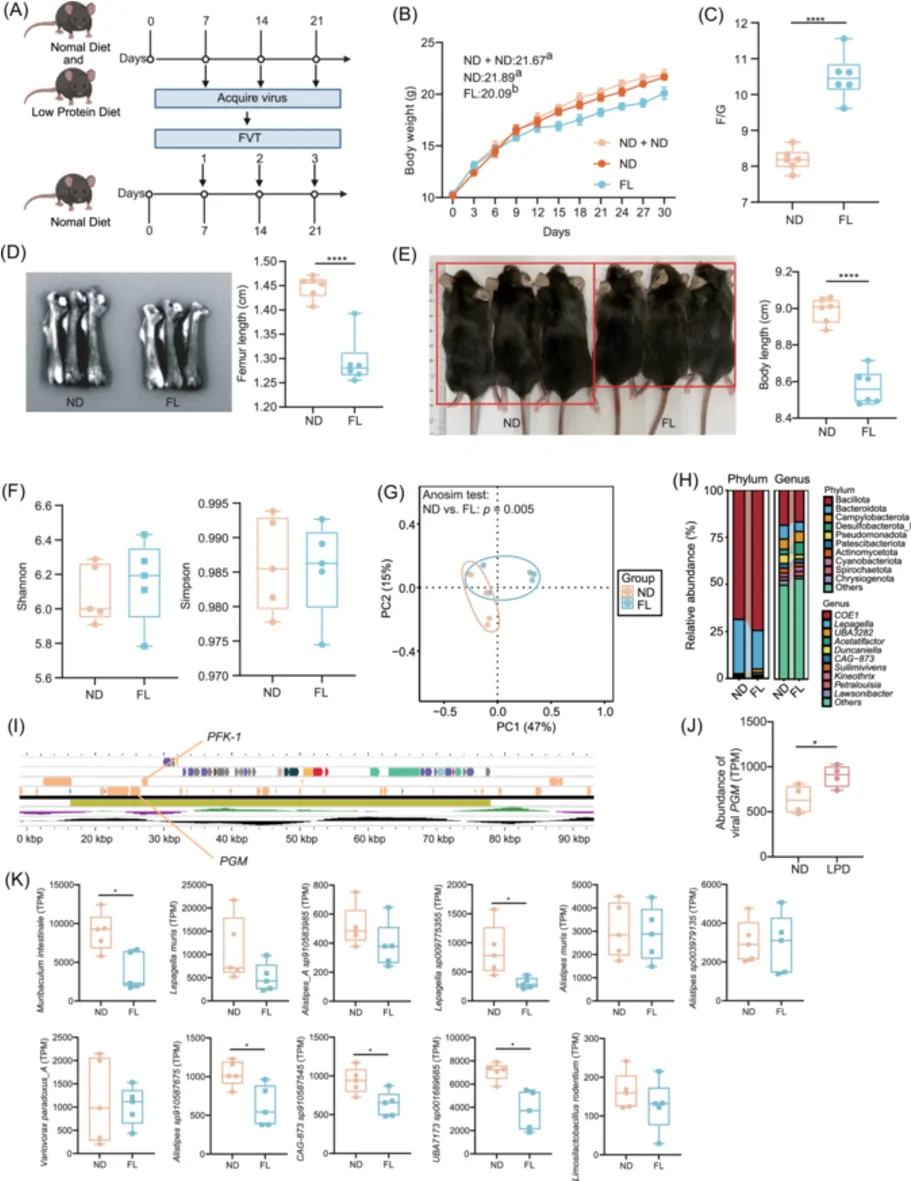
LPD‐derived virome transplantation impairs growth performance and remodels gut microbiota towards carbohydrate metabolism. (A) Schematic illustration of the fecal virome transplantation (FVT) design. Virome extracted from LPD mice was transplanted into ND mice to generate the FL group (FVT‐LPD, FL group). (B–E) Physiological assessment of recipient mice in the FL group compared with ND controls. FL mice recapitulated LPD‐associated growth impairment, including reduced body weight gain (B, *p* < 0.05 for ND vs. FL; *p* > 0.05 for ND vs. ND + ND), increased F/G (C), shortened femur length (D), and reduced body length (E) (n = 6). Gut bacterial community analysis of recipient mice: (F) alpha diversity assessed by Shannon and Simpson indices; (G) PCoA based on Bray–Curtis distances; and (H) taxonomic composition at phylum and genus levels (*n* = 5). (I) Genome map of an integrated prophage region within *Acetatifactor sp011959105*. The prophage encodes metabolic genes associated with glycolytic pathways, including *PGM* and *PFK‐1*. (J) Abundance of *PGM* genes encoded in the ND and LPD viromes (*n* = 4 or 5). (K) Abundance of bacteria harboring complete urease gene clusters in the caeca of ND and FL mice (*n* = 5). For panel B, data are presented as mean ± SEM, final body weight was analyzed using one‐way ANOVA followed by Tukey's post hoc test; different superscript letters indicate significant differences. For panels C, D, E, F, J, and K, data are shown as box plots. Homogeneity of variances was assessed; statistical significance was determined by two‐tailed Student's *t*‐test, and Welch's *t*‐test was applied when variances were unequal (*p < 0.05, ****p < 0.0001).

Metagenomic profiling revealed that LPD virome introduction was associated with alterations in bacterial community composition in recipients. Although gut microbial α‐diversity (Shannon and Simpson indices) remained comparable between the FL and ND groups (*p* > 0.05, Figure 2F), the FL group exhibited a significant shift in overall community composition, as revealed by PCoA analysis (*p* < 0.05, Figure 2G). Notably, bacterial taxa enriched under the LPD condition were also expanded in FL mice, including *Acetatifactor* (Figure 2H). Functionally, the FL microbiome recapitulated LPD‐specific metabolic features: urease maturation and urea metabolism functions were markedly suppressed, coinciding with significantly elevated fecal urea nitrogen (*p* < 0.05), whereas serum urea nitrogen showed no significant difference (Figure S2A,B). Compared with the ND group, the FL group significantly upregulated genes involved in carbohydrate metabolism, degradation, and transport (e.g., fructosamine kinase, glycosyl hydrolases, and ABC transporters), while exhibiting a concomitant reduction in protein and amino acid metabolic capacities. In contrast, the ND group was enriched in genes associated with cell wall modification, protein folding, and structural maintenance (e.g., N‐acetylmuramoyl‐L‐alanine amidase and peptidyl‐prolyl cis–trans isomerase), rather than core carbohydrate metabolic pathways (*p* < 0.05, Figure S2C,D). To determine whether the observed genetic differences were reflected in community‐level metabolic functions, in vitro fermentation assays were performed using starch or carboxymethyl cellulose (CMC) as the sole carbon source. Fermentation with starch showed that transplantation of the LPD virome did not enhance the starch‐degrading capacity of the ND microbiota (Figure S2E). In contrast, CMC fermentation demonstrated that the LPD group exhibited significantly greater cellulolytic activity than both the ND and FL groups. After 12 h of fermentation, the concentrations of reducing sugars released by the LPD and FL groups were both significantly higher than those of the ND group, indicating enhanced early‐stage cellulose degradation (*p* < 0.05, Figure S2E). At 24 h, no significant difference in reducing sugar concentration was observed between the ND and FL groups. By 48 h, however, the ND group retained significantly higher reducing sugar levels than the LPD and FL groups, suggesting that the latter two groups utilized cellulose‐derived sugars more rapidly following their release (*p* < 0.05, Figure S2E). Overall, the LPD group exhibited the strongest capacities for both cellulose degradation and reducing sugar utilization. The FL group displayed an intermediate metabolic phenotype, with greater cellulose degradation and sugar utilization capacities than the ND group but lower than the LPD group, indicating that transplantation of the LPD virome partially enhanced the ability of the ND microbiota to metabolize complex carbohydrates.

Mechanistically, this ecological reshaping is closely linked to phage‐mediated genetic regulation. Metagenome‐assembled genome (MAG) and prophage analyses revealed that *Acetatifactor*, which increased in abundance following FVT, carried a prophage encoding key carbohydrate metabolism AMGs, including phosphoglycerate mutase (*pgm*) and 6‐phosphofructokinase‐1 (*pfkA*) (Figure 2I). Notably, *pgm* gene abundance was higher in the LPD donor virome than in the ND virome (*p* < 0.05), and was concurrently elevated in the FL bacterial metagenome relative to the ND group (*p* < 0.05). This concordant enrichment suggests that phage‐associated AMGs may contribute to the altered carbohydrate metabolism in the bacterial community under LPD conditions (Figures 2J, S2C) [22, 23]. The expansion of carbohydrate‐utilizing bacteria in the FL group suppressed urease‐producing taxa, including *Muribaculum intestinale*, *Lepagella sp009775355*, *Alistipes sp. 910587675*, *CAG‐873 sp. 910587545*, and *UBA7173 sp001689685* (Figure 2K).

To directly evaluate the impact of phage‐driven bacterial metabolic shifts on host nitrogen utilization, we applied a ^15^N‐urea isotopic tracing approach. Two weeks after FVT, FL mice were administered ^15^N‐labeled urea in drinking water, and bacterial‐derived amino acid enrichment in the liver was quantified by LC‐MS (Figure S3A). The liver was chosen as the primary target tissue for isotope enrichment analysis because it serves as a first‐pass metabolic hub for gut‐derived nutrients via the portal vein, allowing capture of microbial metabolites before systemic dilution, unlike peripheral tissues such as muscle or serum. Results demonstrated that LPD virome transplantation substantially impaired microbial nitrogen recycling. In essential amino acids (EAAs), ^15^N incorporation from bacterial urea metabolism into leucine, histidine and methionine was lower in FL mice than in ND controls (*p* < 0.05); lysine labeling was detected only in ND mice, whereas valine appeared exclusively in FL mice (*p* < 0.05, Figure S3B). Among the non‐essential amino acids (NEAAs), only glutamic acid was significantly reduced in the FL group (*p* < 0.05), while the remaining amino acids showed no significant differences (Figure S3B). To determine whether the reduced ^15^N enrichment resulted from decreased bacterial biomass rather than impaired urease activity, we first quantified bacterial abundance in fecal samples from the ND and FL groups. The FL group exhibited significantly lower bacterial abundance than the ND group (*p* < 0.05, Figure S3C). We then inoculated equal numbers of bacteria from the ND and FL groups into urease screening medium. Despite the comparable initial bacterial input, ND‐derived bacteria exhibited faster growth and produced significantly higher levels of extracellular ammonia than FL‐derived bacteria (*p* < 0.05, Figure S3D), indicating reduced urea utilization capacity in the FL bacterial community.

These results demonstrate that alterations in the gut virome are not merely passive consequences of dietary protein restriction, but actively drive host growth limitation. Mechanistically, the LPD‐derived phageome promoted the expansion of carbohydrate‐utilizing bacteria, reduced the abundance of urease‐producing bacteria, and suppressed bacterial utilization of urea nitrogen, thereby decreasing microbial biomass synthesis. Consequently, the host acquired fewer microbially derived amino acids, ultimately resulting in impaired growth.

### ND‐derived virome promote host growth via non‐canonical nitrogen recycling

Given that LPD‐derived virome impair host growth by suppressing nitrogen cycling and locking bacterial metabolic preferences, we hypothesized that introducing ND‐derived virome could reverse this effect. To test this, ND virome was transplanted into LPD mice (FVT‐ND, FN group), with LPD mice receiving LPD virome (LPD + LPD) as negative controls (Figure 3A). Transplantation of LPD‐derived virome into LPD mice had no significant effect on body weight. In contrast, FN treatment elicited a pronounced rescue effect. Compared with LPD controls, FN mice exhibited significantly increased body weight, restored body and femur lengths, and reduced F/G, indicating improved feed efficiency and growth performance (*p* < 0.05, Figure 3B–E).

**FIGURE 3:**
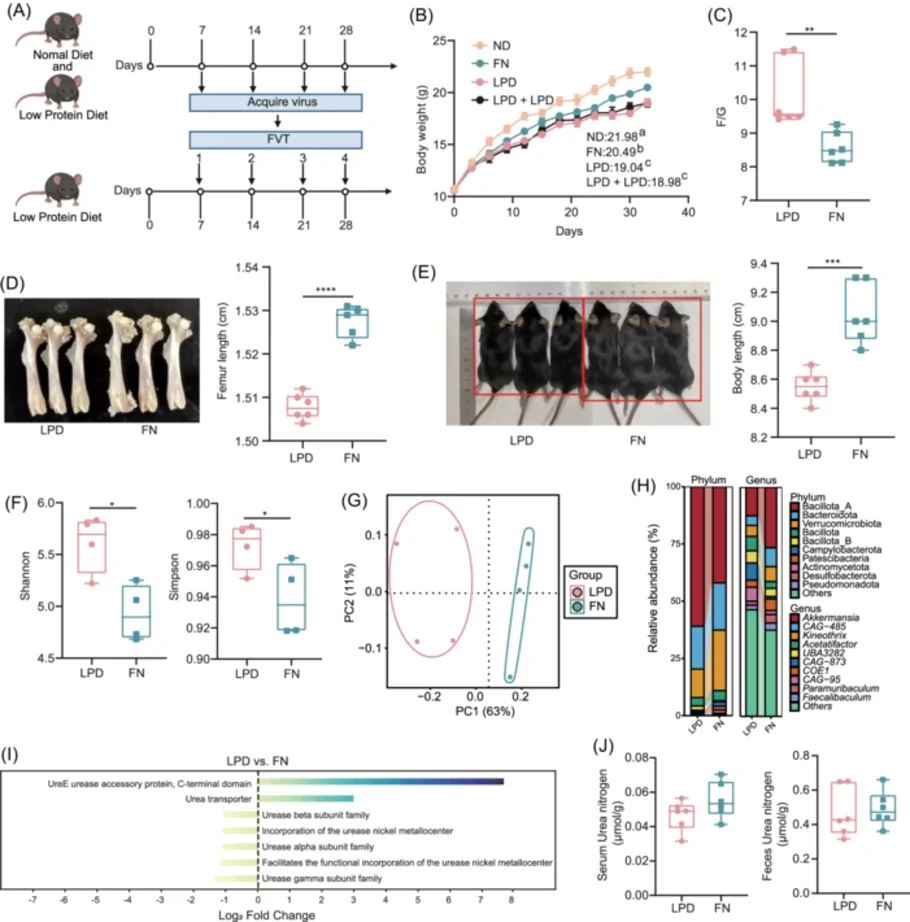
ND‐derived virome transplantation improves growth performance in LPD mice despite limited restoration of microbial urea metabolism. (A) Schematic of the FVT strategy. Virome derived from ND mice was transplanted into LPD mice (FVT‐ND, FN group). (B–E) Growth rescue effects of ND‐derived virome transplantation. FN mice exhibit significantly improved body weight gain (B, *p* < 0.05 for LPD vs. FN; *p* > 0.05 for LPD vs. LPD + LPD; *p* < 0.05 for ND vs. FN), reduced F/G (C), and recovery of femur length (D) and body length (E) compared with LPD controls (*n* = 6). Metagenomic analysis of the gut bacterial community in FN mice: (F) alpha diversity indices (Shannon and Simpson); (G) PCoA based on Bray–Curtis distances; (H) taxonomic composition at the phylum and genus levels (*n* = 4). (I) Differential abundance of genes involved in urea metabolism. Although selected urease accessory genes (e.g., *ureE*) were enriched, genes encoding urease catalytic subunits (*ureA*, *ureB*, and *ureC*) remained low or were not significantly increased in FN mice (*n* = 4). (J) Assessment of host nitrogen status. Serum urea nitrogen (left) shows an increasing trend in FN mice, whereas fecal urea nitrogen (right) remained unchanged and was comparable to LPD levels, consistent with incomplete recovery of intestinal nitrogen recycling efficiency (*n* = 6). For panel B, data are presented as mean ± SEM. For panels C, D, E, F, and J, data are shown as box plots. Statistical significance was determined by two‐tailed Student's *t*‐test; non‐parametric comparisons were performed using the Mann–Whitney *U* test (*p < 0.05, **p < 0.01, ***p < 0.001). For panel I, all features shown are significantly different as determined by DESeq. 2.

Importantly, the rescue effect was not driven by restoration of bacterial diversity. Metagenomic analysis showed that the FN microbiome exhibited significantly lower Shannon and Simpson indices (*p* < 0.05), indicating reduced community diversity and evenness. In addition, β‐diversity differed significantly between groups, demonstrating a marked shift in overall community composition (Figure 3F,G). At both the phylum and genus levels, FN treatment did not restore the microbial composition to that observed in ND mice (Figure 3H).

Functional analyses clarified the direction of this restructuring. Genes associated with highly active simple sugar transport systems (e.g., PTS and TRAP transporters) and protein hydrolysis (e.g., broad‐spectrum peptidases and dipeptidases) that were overrepresented in LPD were significantly downregulated in FN (Figure S4A,B), indicating reduced bacterial dependence on free amino acids and simple carbon sources. Conversely, FN was enriched in enzymes targeting complex non‐starch polysaccharides (NSPs), including cellulases, xylanases, and β‐galactosidases. Notably, GSEA revealed significant upregulation of the “Amino sugar and nucleotide sugar metabolism” pathway (Figure S4C), which synthesizes GlcNAc, UDP‐GlcNAc, and other peptidoglycan precursors [24]. This suggests that FN bacteria may be reallocating metabolic resources toward cell wall repair and regeneration under structural stress [25].

At the nitrogen metabolism level, FN partially restored UreE and urea transport functions, whereas functions associated with core urease catalytic subunits and nickel incorporation remained depleted (Figure 3I), resulting in fecal urea nitrogen levels comparable to those in LPD controls. In contrast, serum urea nitrogen was elevated in FN mice (Figure 3J). This dissociation between fecal urea accumulation and improved host nitrogen status indicates that FN‐mediated growth recovery does not depend on conventional urease‐driven nitrogen recycling, but instead likely involves an alternative, non‐canonical nitrogen pathway.

### ND‐derived phageome exhibits enhanced lytic capacity promoting bacterial protein release

As bacterial functional shifts alone could not fully account for the FN‐induced growth rescue in LPD mice, we explored an alternative phage‐mediated mechanism involving the lytic–lysogenic cycle. Phages classified by replication strategy showed no significant difference in the proportions of lytic and lysogenic types (Figure 4A), indicating that their distinct effects are associated with differences in absolute viral abundance. Quantitative measurements supported this hypothesis that total virus‐like particles (VLPs) absolute abundance in the ND mice was approximately twofold higher than in the LPD mice (Figure 4B) by combining ultracentrifugation (~3 × 10^9^ vs. ~1.5 × 10^9^ particles per gram, respectively) and PEG precipitation (~5 × 10^9^ vs. ~2.8 × 10^9^ particles per gram, respectively) with flow cytometry analysis. Consistently, SYBR Gold staining demonstrated a markedly higher density of VLPs in ND samples (Figure 4C).

**FIGURE 4:**
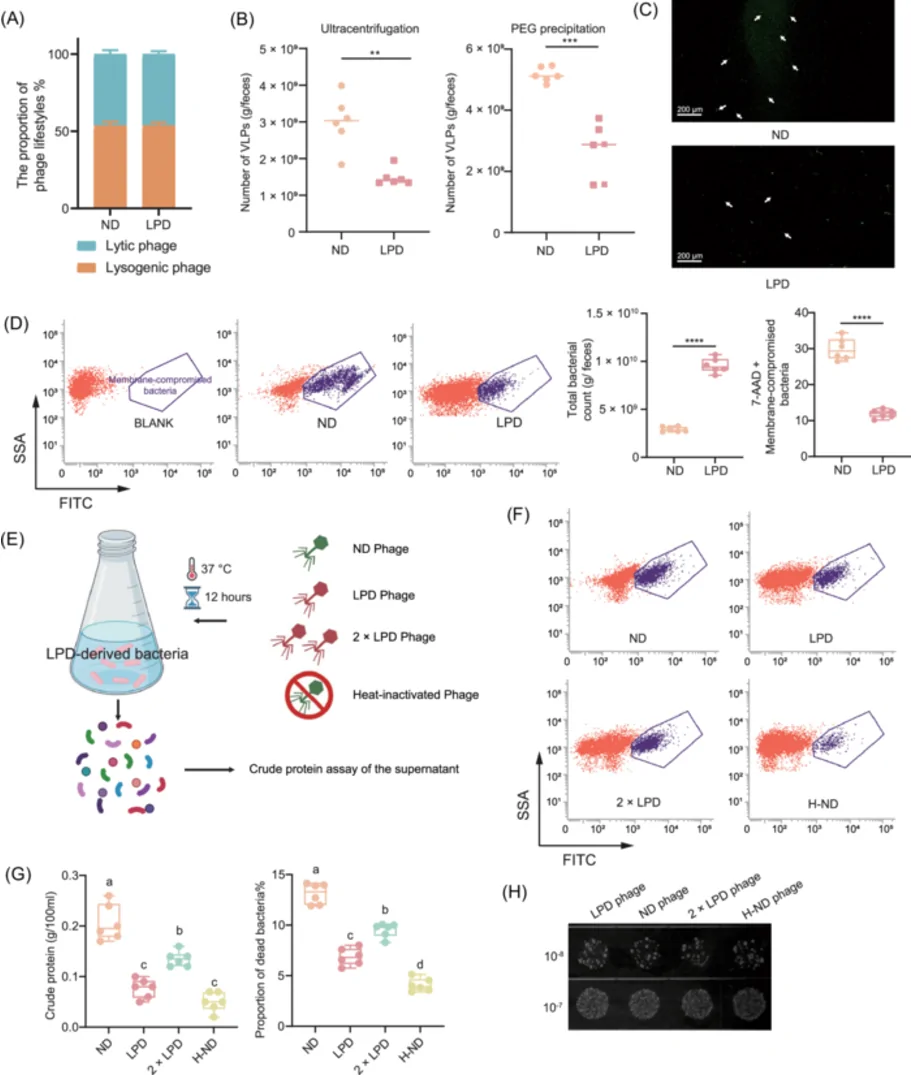
High‐abundance ND‐derived virome exerts strong lytic pressure on LPD‐associated bacteria and promotes bacterial biomass protein release. (A) Proportion of lytic and lysogenic replication strategies (*n* = 4). (B) Fecal virus‐like particle (VLP) abundance measured by ultracentrifugation (left) and PEG precipitation (right), showing higher viral loads in ND mice (*n* = 6). (C) SYBR Gold staining images of fecal samples showing viral particles, with white arrows indicating representative particles. (D) Flow cytometric analysis of fecal bacterial membrane integrity. ND mice exhibited a lower total bacterial load but a higher proportion of membrane‐compromised bacteria compared with LPD mice (*n* = 6). (E) Schematic of the in vitro cross‐infection assay. (F) Flow cytometric assessment of bacterial membrane integrity after cross‐infection, showing increased membrane damage following ND‐derived virome treatment compared with LPD‐derived virome or heat‐inactivated ND‐derived virome controls (H‐ND) (*n* = 6). (G) Crude protein concentration in culture supernatants (*p* < 0.05 for ND vs. LPD; *p* < 0.05 for ND vs. 2×LPD; *p* < 0.05 for 2×LPD vs. LPD) and proportion of membrane‐damaged bacteria (*p* < 0.05 for ND vs. LPD; *p* < 0.05 for ND vs. 2×LPD; *p* < 0.05 for 2×LPD vs. LPD) after cross‐infection, indicating enhanced intracellular protein release in the ND phage group (*n* = 6). (H) Spot plating of serially diluted bacterial cultures after co‐incubation with different phage preparations. For panels D and G, data are shown as box plots. Statistical significance was determined by Student's *t* test; non‐parametric comparisons were performed using the Mann–Whitney *U* test (**p < 0.01, ***p < 0.001, ****p < 0.0001). For panel G, data were analyzed using one‐way ANOVA followed by Tukey's post hoc test; different superscript letters indicate significant differences.

According to the Kill‐the‐Winner framework, elevated phage absolute abundance is expected to impose stronger top‐down control on dominant bacterial populations. To assess the relevance of this mechanism in mediating growth rescue under protein restriction, we evaluated bacterial survival status in vitro lytic potential and in vivo population dynamics. Analysis of bacterial viability showed that, despite a higher total bacterial load in LPD mice, approximately 30% of bacteria in the ND group exhibited compromised membrane integrity (7‐AAD‐positive staining), compared with about 10% in the LPD group (Figure 4D).

In vitro phage release kinetics further supported this interpretation. In a standardized in vitro culture system using GAM–BB medium inoculated with equal fecal bacterial loads from ND and LPD‐fed mice, LPD‐derived phages exhibited a rapid burst of release, exceeding that of ND‐derived phages within the first 60 min of growth (Figure S5A,B). This suggests that LPD bacteria harbor a substantial pool of prophages that remain constrained under protein‐limited conditions but are rapidly induced upon nutrient repletion. To exclude confounding effects of bacterial abundance, equal numbers of bacteria (10^6^ cells) were inoculated into GAM–BB medium, and phage release was quantified during exponential growth, revealing no significant difference between ND‐ and LPD‐derived bacteria (Figure S5C). Further manipulation of nutrient composition showed that ND‐derived bacteria displayed comparable phage release kinetics in GAM–BB and in a nutritionally balanced minimal medium (peptone 12 g/L, glucose 10 g/L), whereas phage release was markedly suppressed under low protein conditions (peptone 3 g/L, glucose 19 g/L) (Figure S5D).

Given that VLP counts alone do not necessarily reflect phage infectivity, we further investigated the mechanisms underlying the growth‐restoring effect of FN in LPD recipients by directly assessing the lytic activity of the ND derived phageome against LPD‐associated bacteria using an in vitro cross‐infection assay. Fecal bacteria isolated from LPD mice were co‐incubated with phage from different dietary origins for 12 h, followed by quantification of bacterial viability and the levels of released bacterial proteins in the supernatant (Figure 4E,F). After 12 h of co‐incubation, cultures treated with heat‐inactivated ND phage showed the fewest membrane‐compromised bacteria among all groups (*p* < 0.05). The ND phage treatment resulted in the highest level of bacterial membrane damage and the highest crude protein content in the supernatant compared with the LPD phage treatment (*p* < 0.05). The 2×LPD group displayed an intermediate response between the ND and LPD phage, with values significantly higher than those of the LPD phage but significantly lower than those of the ND phage (*p* < 0.05, Figure 4G). Spot plating of serially diluted bacterial cultures after co‐incubation with different phage preparations showed distinct growth patterns. Clear bacterial colonies were observed following treatment with ND and 2×LPD phage, whereas slight bacterial lawns remained at the corresponding dilution levels following treatment with heat‐inactivated ND and LPD phage (Figure 4H). Together, these findings indicate that protein limitation reduces the absolute abundance of viral particles by constraining phage release. This leads to higher bacterial loads in the feces of LPD mice. ND‐derived phages exhibited stronger lytic activity against LPD‐associated bacteria, efficiently disrupting bacterial integrity and releasing protein‐rich intracellular contents in an abundance‐dependent manner.

### Viral shunt‐mediated release of bacterial biomass maintains host nitrogen homeostasis

Guided by the in vitro results, ND‐derived and 2×LPD‐derived virome were further tested in vivo by FVT into LPD mice. In addition, LPD mice were co‐housed with recipient (FN) mice, with FL mice serving as negative controls (Figure 5A–C). FN mice exhibited the best growth performance, whereas FL mice showed the poorest growth performance, characterized by significantly lower body weight gain (*p* < 0.05) and higher F/G (*p* < 0.05), with the co‐housing and 2×LPD groups displaying intermediate phenotypes (*p* < 0.05, Figure 5A–C). Body composition analysis revealed no significant differences in fat or lean soft tissue proportions among mice that gained weight in the ND, FN, co‐housing, and 2×LPD groups, indicating that weight gain was not driven by adipose accumulation but rather reflected increased protein acquisition (Figure 5D).

**FIGURE 5:**
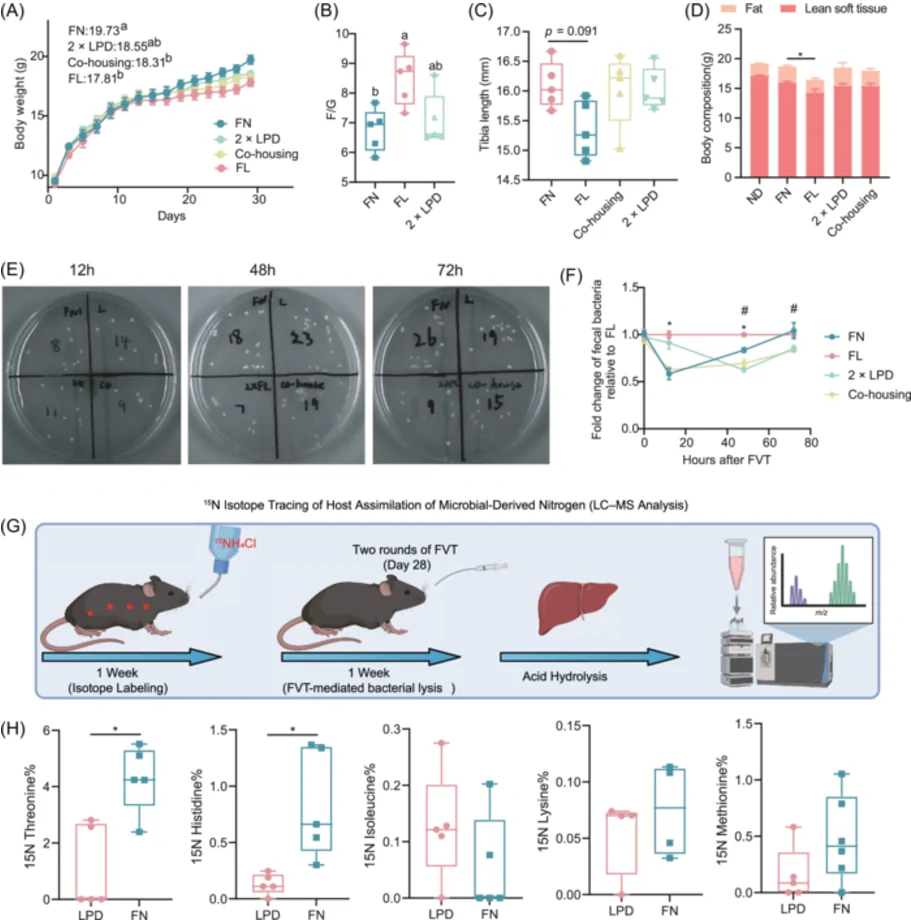
Gut phage‐mediated viral shunt supports host nitrogen acquisition under protein restriction. (A–D) Physiological outcomes of different FVT strategies. (A) Body weight trajectories show that FN mice achieve the most pronounced growth recovery, exceeding co‐housing and 2×LPD phage groups (*p* < 0.05 for FN vs. FL; *p* < 0.05 for FN vs. co‐housing). (B) Comparison of F/G among different FVT treatments (*p* < 0.05 for FN vs. FL; *p* > 0.05 for FL vs. 2×LPD). (C) Tibia length. (D) Body composition analysis indicates comparable lean soft tissue and fat ratios across groups, suggesting that weight gain in FN mice is driven by lean mass rather than fat accumulation (*n* = 5). (E, F) In vivo bacterial lysis dynamics. (E) Representative fecal bacterial culture images at multiple time points post‐FVT. (F) Time‐course quantification of fecal bacterial density relative to LPD baseline (*n* = 6).(G) Schematic of the ^15^N–NH_4_Cl isotope tracing strategy to track bacterial nitrogen flow to the host, bypassing urea recycling pathways. (H) ^15^N‐labeled EAA content in mouse liver (*n* = 5). For panels A and F, data are presented as mean ± SEM. For panels B, C, and H, data are presented as box plots. For panels A–C, statistical significance was evaluated using one‐way ANOVA followed by Tukey's post hoc test; different superscript letters indicate significant differences (*p* < 0.05). For panel D, statistical significance was determined using a two‐tailed unpaired Student's *t*‐test (*p < 0.05). For panel F, at each time point, differences among groups were analyzed using one‐way ANOVA followed by Tukey's post hoc test, with asterisks indicating significant differences between FN and FL groups (*p < 0.05) and hash symbols indicating significant differences between 2× LPD and FL groups (#*p* < 0.05). For panel H, statistical significance was determined using a two‐tailed unpaired Student's *t*‐test, with Welch's correction applied when variances were unequal; non‐parametric comparisons were performed using the Mann–Whitney *U* test (*p < 0.05).

Time‐resolved in vivo analyses yielded compelling support for this interpretation. FN treatment induced a rapid reduction in viable fecal bacteria within 12 h post‐gavage with bacterial abundance decreasing by approximately 50% and gradually recovering over 48–72 h (Figure 5E,F), consistent with predictions of the Kill‐the‐Winner model. In co‐housed mice, bacterial abundance decreased moderately and remained relatively stable, potentially reflecting the acquisition of additional phages through coprophagy. However, coprophagy may also lead to the ingestion of bacterial biomass and microbial proteins, and its contribution to the observed phenotype cannot be determined from the current experiment. In contrast, the 2×LPD virome did not elicit marked bacterial killing at 12 h but induced a delayed reduction in bacterial abundance at 48–72 h, accompanied by partial improvement in host growth (Figure 5E,F). This is attributed to the differential sensitivity of bacterial communities to distinct viromes [26].

To directly test the proposed nutrient flow from phage‐mediated lysis to host assimilation (viral shunt), we employed a ^15^N–NH_4_Cl isotope tracing strategy. This approach avoids confounding effects associated with urease activity and hepatic transamination in ^15^N‐urea experiments. ^15^N–NH_4_Cl can be directly assimilated by gut bacteria for amino acid biosynthesis independent of urease pathways. To minimize host‐derived background, we focused on EAAs that cannot be synthesized de novo by the host and must originate from dietary or microbial sources (Figure 5G). After 1 week of continuous ^15^N–NH_4_Cl labeling, ND virome transplantation was performed, and hepatic amino acid labeling was assessed by LC–MS. Compared with the LPD control group, FN mice showed increased incorporation of microbially derived EAAs, with significant elevations in threonine and histidine (*p* < 0.05, Figure 5H), and non‐significant increases in lysine and methionine (*p* > 0.05, Figure 5H). Together, the ^15^N‐labeling experiments demonstrate that nitrogen derived from ammonium can be incorporated into specific host amino acid pools, while the comparative increase in ^15^N enrichment following ND‐derived virome transplantation, together with enhanced bacterial lysis and extracellular protein release observed in vitro, supports a model in which phage‐mediated bacterial turnover contributes to microbial nitrogen transfer to the host.

## DISCUSSION

PEM remains a primary driver of global childhood stunting and developmental disorders [27]. While gut dysbiosis in malnourished children is well‐documented [8], whether these microecological shifts are passive reflections of host nutritional status or active drivers exacerbating nutrient deficiency has remained a subject of debate. Here, by integrating multi‐omics with ^15^N‐stable isotope tracing, we unveil a central regulatory role for the gut virome in host‐microbe nitrogen partitioning. We show that under low protein stress, phage‐associated metabolic shifts are linked to reduced bacterial urease activity and decreased urea nitrogen recycling, potentially limiting nitrogen availability to the host. Conversely, the introduction of a ND‐derived virome liberates this nitrogen via bacterial lysis for host utilization. These findings challenge the conventional view of enzymatic mutualism, proposing instead a non‐canonical nitrogen compensation mechanism based on ecological predation.

Consistent with our findings, LPD has been reported to reduce intestinal urease activity [7]. In our study, LPD increased the abundance of carbohydrate‐utilizing bacteria, accompanied by a reduction in ureolytic taxa and enrichment of phages carrying carbohydrate metabolism‐associated AMGs, including *pgm*. Following virome transplantation into ND mice, the expansion of carbohydrate‐utilizing taxa, particularly *Acetatifactor*, was also observed. Notably, genomic analysis revealed that *pgm* was located within a prophage region of *Acetatifactor*, suggesting that prophage‐associated metabolic potential may contribute to enhanced carbohydrate utilization and ecological adaptation of this bacterial population under protein‐limited conditions. The expansion of carbohydrate‐utilizing bacteria was accompanied by reduced ureolytic bacterial abundance and impaired nitrogen recycling, ultimately contributing to decreased host nitrogen recovery efficiency and growth performance.

Notably, host recovery occurred independently of restored urea recycling functions. In phenotypically recovered mice, core urease gene abundance remained static, and fecal urea nitrogen levels remained elevated. Instead,^15^N ‐tracing and flow cytometry analyses reveal that high‐abundance ND phages execute a “Kill‐the‐Winner” strategy [28]. This mechanism curbs bacterial overgrowth and simultaneously acts as a “gut viral shunt,” lysing dominant bacteria to release proteins and amino acids sequestered within them for host utilization [29, 30]. This suggests that under nutritional scarcity, nitrogen acquisition via biomass predation is a more direct and efficient strategy than reconstructing complex enzymatic cooperation.

Mechanistically, the indigenous LPD phageome may be unable to initiate such compensatory lysis under energetic constraints, as phage replication and release depend on host bacterial protein synthesis. In the energy‐limited LPD environment, phages favor lysogeny [31], integrating into the bacterial genome and expressing AMGs to ensure mutual survival. Furthermore, when transplanted into protein‐sufficient hosts, the LPD phageome failed to induce lysis due to unique ecological constraints. First, the viral load was insufficient to reach the density threshold required to trigger “Kill‐the‐Winner” cycles [32]. Second, specific LPD‐associated taxa (e.g., *Acetatifactor*) occupied a “predation‐free” niche—evading both the low‐density introduced phages and host‐specific indigenous phages. Consistent with this mechanism, FVT derived from 2×LPD enhanced growth performance in LPD mice and was associated with increased fecal bacterial lysis. The release of predation pressure, together with phage‐associated metabolic shifts, enables carbohydrate‐utilizing taxa to competitively exclude ureolytic symbionts, thereby reinforcing a nitrogen‐locking metabolic state.

Notably, although equivalent amounts of LPD‐ and ND‐derived VLPs were transplanted, the LPD‐derived phageome exhibited reduced bacteriolytic activity, indicating that VLP abundance alone does not necessarily reflect functional infectivity. Nevertheless, under conditions of nutritional recovery, LPD‐associated bacteria still released large amounts of phages, suggesting a functional shift in viral infection dynamics under a low‐protein environment. This pattern may be explained by two non‐mutually exclusive mechanisms. First, the LPD environment suppresses phage induction and release, favoring a lysogeny‐associated functional state and limiting productive lytic infection. Second, nitrogen limitation may impair phage assembly and maturation [33], resulting in structurally defective or poorly infectious virions that are still detected as viral particles but exhibit reduced infectivity. By contrast, ND‐derived phages may be more efficiently assembled under nutrient‐replete conditions, potentially maintaining higher particle integrity and lytic activity, which could contribute to enhanced host lysis and subsequent release of host‐derived proteins. However, this hypothesis requires further validation through direct assessments of viral particle morphology and integrity, such as transmission electron microscopy, as well as functional infectivity assays.

In summary, we identify the gut virome as a critical arbiter of host‐microbe nitrogen partitioning. By manipulating viral‐bacterial interactions to shift the ecosystem from metabolic sequestration to lytic release, host nitrogen status can be improved with or without altering dietary intake. This finding not only redefines the pathophysiological role of the microbiome in malnutrition but also informs novel clinical interventions. Although our study demonstrates the efficacy of FN in improving host growth performance, the direct implementation of whole‐virome transplantation faces substantial translational barriers. These include potential biosafety concerns, such as the transfer of eukaryotic viruses or antibiotic resistance genes, as well as the absence of unified and controllable standardization procedures. A defining feature of the LPD gut ecosystem is the excessive accumulation of bacterial biomass accompanied by suppressed phage activity, resulting in a pronounced ecological imbalance. The core mechanism underlying this imbalance is the sequestration of nitrogen within biomass by bacteria that have effectively escaped viral predation, thereby limiting the host's capacity to utilize nitrogen efficiently. In light of these findings, future intervention strategies need not focus on replacing the entire virome. Instead, they should aim to restore phage–bacteria lytic interactions in situ to release nitrogen trapped within microbial nutrient reservoirs. Our in vitro findings raise the possibility that LPD‐associated bacteria harbor dormant prophages that could be induced into the lytic cycle under appropriate nutritional conditions. This observation generates the testable hypothesis that dietary components, including d‐xylose, short‐chain fatty acids, and selected food‐derived polyphenols, may promote endogenous prophage induction, a possibility that warrants further investigation in vivo [34, 35, 36]. Such activation could convert bacterial biomass into host‐accessible amino acids without the need for exogenous viral introduction. Alternatively, to overcome deficiencies in particle integrity and infectivity within the indigenous LPD phageome, a more direct strategy would involve the administration of engineered phage cocktails. Unlike broad‐spectrum ND phageome, these cocktails could be specifically formulated to target dominant “nitrogen‐sequestering” taxa identified in this study. In addition, quorum‐quenching agents may serve as complementary interventions by perturbing bacterial quorum sensing and metabolic states, which weaken collective antiviral defenses and promote phage infection and lytic activity [37, 38, 39, 40].

Although this study was conducted in a mouse model, whether similar bacterial and phage population dynamics occur in other host species under protein restriction remains to be determined. Prophages were identified using stringent genome‐based approaches; however, computational predictions alone are insufficient to resolve their induction status, infectivity, or functional contribution. In particular, although the identification of *pgm* and *pfkA* within an *Acetatifactor*‐associated prophage suggests potential metabolic functions encoded by phages, their direct contribution to bacterial metabolic phenotypes remains to be experimentally validated. Future studies combining prophage induction assays, heterologous expression of candidate AMGs, and genetically defined phage–host systems will be required to establish the causal roles of phage‐encoded genes in regulating bacterial metabolism. Furthermore, we were unable to experimentally induce prophage activation using mitomycin C. This limitation may reflect the technical challenges of reproducing prophage induction in complex gut microbial communities, where many bacterial hosts are difficult to culture, induction responses vary across host taxa, and only a subset of temperate phages are responsive to DNA damage. Isotope tracing based on liver tissue measurements provides evidence consistent with the incorporation of microbiota‐derived nitrogen into host amino acid pools but does not directly quantify nitrogen fluxes or resolve intermediate processes such as microbial ammonium assimilation and bacterial protein turnover. Thus, our findings support, but do not directly establish, a complete pathway linking microbial nitrogen assimilation, phage‐mediated bacterial lysis, and subsequent host nitrogen uptake. Future studies using gnotobiotic models, defined phage–host consortia, or targeted phage genetic manipulation will be required to further dissect the contribution of specific bacteriophages to microbial nitrogen transfer and host protein utilization. Despite these limitations, our findings support a model in which the gut virome acts as an active regulator of bacterial community structure, nutrient flux, and host protein utilization rather than a passive responder to dietary changes.

## CONCLUSION

In conclusion, our findings reveal a dual role of the gut phageome in regulating host nitrogen metabolism. Under normal conditions, phage‐mediated bacterial lysis facilitates microbial protein release and sustains nitrogen recycling to the host. Conversely, dietary protein restriction reshapes phage–bacterial interactions and microbial metabolism, impairing bacterial nitrogen utilization and ultimately weakening host nitrogen metabolism.

## METHODS

### Animals

All animal experiments were conducted in strict accordance with the Chinese guidelines for the welfare and ethical review of laboratory animals and were approved by the Animal Welfare Committee of the College of Animal Science, Zhejiang University (Approval No. ZJU20230156). Three‐week‐old male C57BL/6J mice were purchased from Hangzhou Medical College. Mice were housed in a barrier system under controlled conditions, with a constant temperature of 22 ± 2°C, relative humidity of 55 ± 5%, and a 12 h light/12 h dark cycle. Animals had ad libitum access to food and water. The composition of the LPD diet is provided in Table S1. During the experimental period, fecal samples were collected daily for subsequent analyses.

### Enrichment and purification of virus‐like particles

Fecal and intestinal content samples were weighed, and their dry weight was recorded. Five volumes of SM buffer (8 mmol/L MgSO_4_, 100 mmol/L NaCl, 50 mmol/L Tris‐HCl, pH 7.4, 0.002% w/v gelatin) were added to each sample, and the mixtures were vortexed and manually homogenized on ice until fully homogenized. Homogenates were centrifuged at 4°C at 4000 × *g* for 5–10 min to remove large debris, and the supernatants were transferred to new tubes and centrifuged again under the same conditions. The resulting clarified supernatants were further centrifuged at 13,500 × *g* for 5–10 min at 4°C to further deplete residual bacterial cells and cellular debris, reducing bacterial contamination and the carryover of non‐viral particles [41]. Finally, the supernatants were sequentially passed through 0.45 and 0.2 μm syringe filters to remove bacteria and eukaryotic cells, as described in established intestinal virome protocols [42]. The resulting supernatants were subjected to downstream processing as required for subsequent analyses.

VLPs were concentrated by sucrose cushion ultracentrifugation. Filtered supernatants were transferred to ultracentrifuge tubes containing a 28% (w/w) sucrose cushion and centrifuged at 160,000 × *g* for 2 h at 4°C. The supernatant was discarded, and the pellet was resuspended in an equal volume of SM buffer. Samples were then treated with DNase I (20 U/mL) and incubated at room temperature for 1 h to remove free nucleic acids, followed by heat inactivation of the enzyme at 65°C for 15 min. After a brief centrifugation at 2000 rpm for 5 min, the resulting supernatant was collected for nucleic acid extraction [43].

For samples used in FVT, VLPs were concentrated using polyethylene glycol (PEG) precipitation. PEG 8000 was added to the filtered supernatant to a final concentration of 20% (w/v) together with NaCl at 0.75 M, and samples were incubated on ice overnight. Samples were then centrifuged at 3270 × *g* for 1 h at 4°C. The supernatant was discarded, and the pellet was resuspended in SM buffer. A second precipitation was performed by adding 5× PEG solution, incubating on ice for 30 min, and centrifuging at 27,000 × *g* for 15 min. The final pellet was resuspended in SMN buffer (SM buffer mixed with 1 mol/L NaHCO_3_ at a 10:2 ratio). Subsequently, 0.3 volumes of chloroform were added, gently mixed, and centrifuged at 2500 × *g* for 5 min. The aqueous phase was then incubated with DNase I at room temperature for 1 h to remove free nucleic acids, followed by heat inactivation of the enzyme at 65°C for 15 min. PEG precipitation has been widely applied for gut virome extraction and generates viral particle preparations suitable for downstream analyses [44].

### FVT and experimental design

Three‐week‐old male SPF C57BL/6J mice were randomly assigned to the control or experimental groups and fed their respective diets (ND or LPD). After 1 week of dietary intervention, the mice underwent FVT. FVT was administered by oral gavage, with each mouse receiving virome preparations equivalent to 50 mg of donor fecal material per treatment. This corresponded to approximately 2.5 × 10^8^ VLPs/mouse for ND‐derived virome and 1.4 × 10^8^ VLPs/mouse for LPD‐derived virome. The 2×LPD group received virome preparations equivalent to 100 mg of LPD fecal material (~2.8 × 10^8^ VLPs/mouse). For LPD‐FVT experiments, mice received FVT once weekly for 3 consecutive weeks. For ND‐FVT experiments, transplantation was performed once weekly for 4 consecutive weeks. The different transplantation durations were determined according to the experimental timeline and downstream phenotypic analyses of each study. During the experimental period, mouse body weight, daily food intake, and F/G were continuously monitored. The ND‐FVT and LPD‐FVT experiments presented in Figures 2 and 3 were independently repeated three times as complete animal experiments. Because each experiment was performed as an independent animal cohort, representative data from one experiment are shown, while consistent results across experiments confirmed reproducibility.

### Viral and metagenomic sequencing and bioinformatics analysis

Total viral DNA was extracted from enriched VLPs using the E.Z.N.A.® Viral DNA Kit (Omega Bio‐tek, Norcross, GA, USA). Total bacterial DNA from intestinal contents was extracted using the E.Z.N.A.® Stool DNA Kit (Omega Bio‐tek) following the manufacturer's protocols. DNA samples that passed quality control (OD260/280 between 1.8 and 2.2) were used for library construction with the TruSeq™ Nano DNA Sample Preparation Kit (Illumina, San Diego, CA, USA), targeting approximately 400 bp insert size. Paired‐end sequencing (PE150) was performed on an Illumina NovaSeq. 6000 platform (Illumina). Initial read quality control, adapter trimming, and host contamination removal were performed using Trimmomatic (SLIDINGWINDOW:4:15, MINLEN:75) prior to de novo assembly with MEGAHIT (‐‐min‐contig‐len 500) to generate contigs from the clean reads.

### Bacterial community analysis

Taxonomic classification and relative abundance estimation of bacterial communities were obtained using Kraken2 with a comprehensive reference database, followed by abundance refinement using Bracken. Kraken2 assigns sequencing reads based on exact k‐mer matches, and Bracken uses a Bayesian approach to provide more accurate species‐level abundance estimates from Kraken2 outputs [45].

### Viral community analysis

Raw reads were first mapped to the host reference genome using BWA‐MEM (v0.7.17), and host‐derived reads were removed. The remaining non‐host reads were assembled de novo using MEGAHIT, and contigs longer than 2000 bp were retained for downstream virome analysis. Candidate viral contigs were identified using four complementary approaches: DeepVirFinder (score ≥ 0.7, *p* < 0.05) [46], VirSorter2 (score ≥ 0.7) [47], VIBRANT with its built‐in bacterial gene filtering algorithm, and sequence similarity searches against the IMG/VR database (*E*‐value ≤ 1 × 10^−5^). To further improve specificity, candidate viral contigs were subsequently classified using geNomad, and contigs predicted as bacterial chromosomal sequences or chromosome‐associated elements were discarded. This two‐step identification strategy, combining consensus viral prediction with secondary genome classification, minimized bacterial contamination in the final viral dataset. High‐confidence viral contigs were clustered into viral operational taxonomic units using MUMmer based on ≥95% average nucleotide identity and ≥85% alignment coverage [48].

Open reading frames (ORFs) within viral contigs were predicted using METAProdigal [49]. Functional annotation of predicted proteins was performed with KofamScan against the KEGG database and DIAMOND searches against the CAZy, eggNOG, and NCBI NR databases to assign potential metabolic functions. Host prediction and taxonomic assignment for viral sequences were conducted using VPF‐Class, which integrates viral family‐specific features and known host associations.

### Identification and functional analysis of prophages based on MAGs

Based on MAGs, potential viral sequences were identified using VirSorter2 [47] and assessed for quality with CheckV [50, 51]. Only sequences classified as category 1 or 2 by VirSorter2 and further confirmed as viral by VIBRANT (‐virome mode) were retained [52]. Prophages were identified as viral regions that were annotated as “Integrated prophages” by CheckV and predicted by PHASTEST with a confidence score >80. ORFs of the prophages were predicted using MetaProdigal, and their auxiliary metabolic functions were analyzed by DIAMOND BLASTP (e‐value < 1e‐5) searches against the COG and KEGG databases.

### Assessment of bacterial carbon source utilization via in vitro fermentation

This study employed a modified M9 minimal medium as the basal culture system to evaluate the capacity of gut microbiota from different treatment groups to utilize a single carbon source. The M9 medium consisted of Na_2_HPO_4_ (6.8 g/L), KH_2_PO_4_ (3.0 g/L), NaCl (0.5 g/L), and NH_4_Cl (1.0 g/L), supplemented with yeast extract (0.5 g/L) to provide trace growth factors required by gut microorganisms. After sterilization, MgSO_4_ (2 mM) and CaCl_2_ (0.1 mM) were added [53]. Two culture systems were established using 0.5% (w/v) soluble starch or 0.5% (w/v) carboxymethyl cellulose (CMC) as the sole carbon source to assess microbial utilization of readily degradable carbohydrates and structural polysaccharides. Fecal samples were prepared as 1% (w/v) suspensions, inoculated (10% v/v) into each medium, and incubated anaerobically at 37°C [53]. Sampling schedules were designed according to substrate degradation characteristics: samples were collected at 0, 6, 12, and 24 h for the starch group, and at 0, 6, 12, 24, and 48 h for the CMC group. To remove bacterial pellets, the fermentation broth was centrifuged at 12,000 rpm for 5 min, and the resulting supernatants were collected for metabolic analysis. Starch residues were quantified using the iodine–potassium iodide (I_2_–KI) colorimetric method [54], while cellulose degradation capacity was assessed by measuring reducing sugar release using the 3,5‐dinitrosalicylic acid (DNS) assay [55].

### ^15^N isotope tracing analysis in liver tissue

To determine whether the FL virome reduces intestinal urea nitrogen recycling and thereby impairs host growth, we evaluated microbial urea nitrogen utilization using a ^15^N‐urea tracing approach. All mice were maintained on a normal diet, with the ND group receiving no treatment and the FL group receiving two FVT derived from LPD mice. After two FVT administrations at 1‐week intervals, ^15^N‐urea was administered through drinking water (1 mg/mL, 99 atoms% ^15^N). Following 1 week of continuous intake, liver samples were collected for analysis of microbiota‐derived nitrogen incorporation.

To determine whether ND‐FVT increases the uptake of bacterial biomass‐derived proteins by the host, we traced microbial nitrogen metabolism using a ^15^N‐ammonium chloride labeling approach. All mice were fed a LPD and provided with drinking water containing ^15^N‐ammonium chloride (1 mg/mL, 99 atoms% ^15^N) ad libitum. After 1 week of labeling, mice in the FN group received FVT every 3 days, with viromes derived from normally fed mice. Following two FVT administrations, mice were euthanized and liver samples were collected for analysis of ^15^N incorporation.

20 mg of freeze‐dried liver powder was accurately weighed and placed into a 15 mL Chemical Oxygen Demand digestion tube. One milliliter of 6 M hydrochloric acid was added, and the tube was flushed with nitrogen gas and sealed. Samples were hydrolyzed at 120°C for 6 h. After cooling, nitrogen gas was used to evaporate the acid to dryness. The residue was re‐dissolved in 50 μL of 80% acetonitrile in water, vortexed thoroughly, and then 450 μL of 0.1 M sodium acetate solution was added [56]. The mixture was vortexed again and filtered before transferring to an injection vial. Samples were analyzed using an Agilent 1290 Infinity II UPLC coupled with a Bruker timsTOF Pro2 mass spectrometer. The chromatographic column was an ACQUITY UPLC BEH Amide (100 mm × 2.1 mm, 1.7 μm). Mobile phase A consisted of water containing 15 mM ammonium acetate and 0.3% ammonia, and mobile phase B consisted of acetonitrile:water (9:1, v/v) with the same additives. The flow rate was 0.3 mL/min, and the gradient program was as follows: 0–1 min, 95% B; 1–9 min, linear decrease to 50% B; 9–12 min, maintained at 50% B; 12–12.5 min, increased to 95% B and equilibrated until 14 min. The injection volume was 2 μL. Mass spectrometry was performed in positive electrospray ionization (ESI) mode with a scanning range of m/z 20–1300. Parameters were set as follows: capillary voltage 4500 V, nebulizer gas 2 bar, drying gas 8 L/min at 230°C, and sheath gas 4 L/min at 400°C. The relative abundance of ^15^N in liver amino acids was measured to evaluate host assimilation of microbiota‐derived nitrogen.

### Urea determination

Urea nitrogen concentrations in feces and serum were determined using a urease‐based colorimetric assay according to the manufacturer's instructions (Nanjing Jiancheng Bioengineering Institute, Nanjing, China). Fecal samples were diluted 10‐fold with PBS, mixed with 0.25 mL of urease buffer, and incubated at 37°C for 10 min. Subsequently, 1 mL of the phenol chromogenic reagent and alkaline sodium hypochlorite solution was added, followed by incubation in a water bath for 10 min. Absorbance was measured at 640 nm. Urea nitrogen concentrations were calculated according to the formula provided in the manufacturer's instructions. Serum samples were analyzed directly without dilution using the same assay.

### Urease activity assay

To assess the urea utilization capacity of bacterial communities from the ND and FL groups, fecal bacterial suspensions were normalized to the same initial inoculum and separately inoculated into a urea‐containing medium. The medium contained peptone (1 g/L), NaCl (5 g/L), glucose (1 g/L), and KH_2_PO_4_ (2 g/L). After autoclaving, filter‐sterilized urea solution was added to a final concentration of 20 g/L. The cultures were incubated statically under anaerobic conditions at 37°C for 36 h. Bacterial growth was monitored by measuring OD_600_ every 6 h. After 36 h of incubation, culture supernatants were collected to quantify extracellular ammonia. Briefly, 20 μL (0.02 mL) of culture supernatant was mixed with 1 mL of phenol reagent and 1 mL of alkaline sodium hypochlorite solution. After thorough mixing, the samples were incubated at 37°C for 10 min to allow color development. Absorbance was then measured at 640 nm as an indicator of extracellular ammonia production.

### Total nitrogen determination

Total nitrogen in bacterial culture supernatants was determined using the Kjeldahl method [57]. Five milliliters of culture supernatant were accurately measured and mixed with digestion reagents (0.4 g CuSO_4_ and 6 g K_2_SO_4_) and 20 mL concentrated sulfuric acid. Samples were pre‐digested at 200°C until charring occurred, then heated to 450°C until the solution became clear blue‐green and maintained for 1 h. After cooling, the digest was diluted with water and distilled under alkaline conditions, with released ammonia absorbed by boric acid solution. Total nitrogen content was calculated by titration with standardized sulfuric acid using methyl red–bromocresol green as the indicator, based on the volume of acid consumed.

### Viral quantification

Isolated intestinal viral particles were stained with SYBR Gold nucleic acid dye and incubated at 37°C for 1 h. The stained viral particles were enumerated by flow cytometry for absolute quantification. Each sample was analyzed for 12 s, and the recorded event counts were converted to particle concentrations based on the analyzed sample volume. A subset of samples was also examined by fluorescence microscopy with whole‐field fluorescence scanning to visualize SYBR Gold‐positive signals.

### Bacterial activity and in vivo lysis stress assessment

Fresh fecal samples were collected from mice and resuspended in PBS with thorough vortexing. Large fibrous debris was removed by passing the suspension through a 45 μm cell strainer, and viral particles in the supernatant were removed by centrifugation at 3000 × *g* for 10 min. The resulting bacterial pellet was washed three times with sterile PBS to remove residual contaminants, and the cleaned bacterial pellet was retained for subsequent analyses. Total bacterial populations were first gated using SYBR Gold nucleic acid dye by flow cytometry. Dead bacteria were subsequently identified by 7‐AAD (7‐Aminoactinomycin D) staining, and the proportion of 7‐AAD‐positive cells was determined to quantify in vivo bacterial lysis stress.

### *In vitro* phage release and cross‐infection experiments

For phage release kinetics, bacteria isolated from the feces of ND and LPD mice were inoculated into GAM‐BB medium and incubated at 37°C. Samples were collected at different time points to quantify phage release. The effects of normal‐ and low‐protein nutritional conditions on phage release were also evaluated. Normal‐protein conditions were established using a defined minimal medium containing peptone (12 g/L) and glucose (10 g/L), whereas low‐protein conditions were established using a defined medium containing reduced peptone (3 g/L) and elevated glucose (19 g/L).

For cross‐infection experiments, cecal bacteria from LPD mice were used as the substrate and co‐incubated at 37°C for 12 h with ND viral communities, LPD viral communities, twofold‐concentrated LPD viral communities, or heat‐inactivated ND viral communities in SM buffer supplemented with 10% BHI [58]. The following parameters were measured: total protein content in the supernatant as an indicator of bacterial lysis, the proportion of dead bacteria determined by flow cytometry, and bacterial abundance assessed by spot plating assays.

### In vivo monitoring of bacterial dynamics

Fecal samples were collected from mice at 0, 12, 48, and 72 h after viral community transplantation. Samples were serially diluted, and aliquots of the appropriate dilutions from the FN, FL, 2×LPD, and co‐housed FN groups were simultaneously plated onto separate designated areas of the same agar plate. The plates were incubated overnight at 37°C under anaerobic conditions. Following incubation, colony‐forming units (CFUs) were counted to quantify culturable viable bacteria. The CFU counts were normalized to those of the FL group at each time point, which was used as the reference group, to assess temporal changes in culturable viable bacterial abundance following viral community transplantation.

### Quantification and statistical analysis

Data were tested for normality and homogeneity of variance prior to statistical comparisons using the Shapiro–Wilk and Levene's tests, respectively. Statistical analyses were conducted using GraphPad Prism 10. Data were presented as means ± SEM. Statistical comparisons between two groups were performed using an unpaired Student *t*‐test or Mann–Whitney *U* test. Statistical comparisons between more than two groups were performed with one‐way ANOVA with Tukey's correction. Statistical details of experiments can be found in the figure legends.

## AUTHOR CONTRIBUTIONS

**Shujie Xu**: Conceptualization; investigation; writing—original draft; writing—review and editing; visualization; methodology; validation; formal analysis. **Xianglin Fei**: Methodology; validation. **Fengqing Lin**: Methodology; validation; writing—review and editing. **Wenzi Wu**: Methodology. **Jiachen Zhuang**: Validation. **Qiufen Mo**: Writing—review and editing. **Xiuan Zhan**: Funding acquisition. **Aikun Fu**: Funding acquisition; conceptualization; writing—review and editing; supervision; project administration.

## CONFLICT OF INTEREST STATEMENT

The authors declare no conflicts of interest.

## ETHICS STATEMENT

All animal experiments were conducted in strict accordance with the Chinese guidelines for the welfare and ethical review of laboratory animals and were approved by the Animal Welfare Committee of the College of Animal Science, Zhejiang University (Approval No. ZJU20230156).

## Supporting information


**Figure S1:** LPD remodels the gut bacteriome towards carbohydrate metabolism and reduced nitrogen metabolism capacity.
**Figure S2:** LPD‐derived virome transplantation reshapes microbial metabolic functions and alters host nitrogen status.
**Figure S3:** ^15^N‐urea isotope tracing reveals reduced host assimilation of microbiota‐derived nitrogen following LPD‐derived virome transplantation.
**Figure S4:** ND‐derived virome transplantation does not restore LPD‐associated bacterial metabolic functions.
**Figure S5:** Protein availability dictates phage release kinetics and lytic activity.

**Table S1:** Composition and nutrient levels of ND and LPD.

## Data Availability

All data generated in this study are publicly available under NCBI BioProject accession number PRJNA1420495 (https://www.ncbi.nlm.nih.gov/bioproject/PRJNA1420495). The data and scripts used are saved in GitHub (https://github.com/154749642-coder/Xu2026iMeta). Supplementary materials (figures, tables, graphical abstract, slides, videos, Chinese translated version, and updated materials) can be found in the online DOI or iMeta Science http://www.imeta.science/.
