# Supplementary material for: Gut virome orchestrates nitrogen partitioning between the host and microbiome
Source: Imeta. 2026 Oct 4;5(5):e70177. doi: 10.1002/imt2.70177 (PMC13635721; doi:10.1002/imt2.70177)
Supplement: Supplementary file 1 [file IMT2-5-e70177-s002.docx]

**Supporting information to**

# **Gut virome orchestrates nitrogen partitioning between the host and microbiome**

**Running title**: Gut virome regulates nitrogen salvage

Shujie Xu^1^; Xianglin Fei^1^; Fengqing Lin^2^; Wenzi Wu^1^; Jiachen Zhuang^1^; Qiufen Mo^3^; Xiuan Zhan^1^; Aikun Fu^1^*

^1^ Zhejiang Key Laboratory of nutrition and breeding for high-quality animal products, College of Animal Sciences, Zhejiang University, Hangzhou 310058, China

^2^ Department of Critical Care Medicine, Jinling Hospital, Affiliated Hospital of Medical School, Nanjing University, Nanjing 210002, China

^3^ National Key Laboratory for Development and Utilization of Forest Food Resources, College of Food and Health, Zhejiang A&F University, Hangzhou 311300, China

* Correspondence: aikunfu@zju.edu.cn (Aikun Fu)

**Figure S1 Low-protein diet (LPD) remodels the gut bacteriome towards carbohydrate metabolism and reduced nitrogen metabolism capacity.**
(A) Alpha diversity (Shannon and Simpson indices) of the bacterial community (n = 4 or 5).
(B) PCoA based on Bray–Curtis distances (n = 4 or 5).
(C) Taxonomic composition of the gut bacteriome at the phylum (left) and genus (right) levels (n=4 or 5).
(D) Heatmap and hierarchical clustering of CAZymes (n = 4 or 5).
(E) Differential abundance of genes involved in urease biosynthesis and nitrogen metabolism (n=4 or 5).

Data in panel A are shown as box plots. Statistical significance was assessed using a two-tailed Student’s t-test. The heatmap in panel D shows TPM values after Z-score transformation. Differential abundance analyses in panel E were performed using DESeq2.

**Figure S2 LPD-derived virome transplantation reshapes microbial metabolic functions and alters host nitrogen status.**
(A) Differential abundance of microbial genes related to urea metabolism between normal diet (ND) and FL groups. Genes required for urease maturation and catalytic subunits are significantly depleted in the FL microbiome, indicating impaired microbial nitrogen recycling capacity (n = 5).
(B) Physiological assessment of host nitrogen status. Serum urea nitrogen (left) and fecal urea nitrogen (right) concentrations in ND and FL mice (n = 6).
(C) Differential abundance of genes involved in carbohydrate metabolism (n = 5).
(D) Differential abundance of genes related to protein and amino acid metabolism (n = 5).

(E) Upper panel shows the cellulolytic activity of different bacterial fractions as assessed by CMC degradation; lower panel shows their amylolytic activity as assessed by soluble starch degradation (n = 5).

Data in panel B are shown as box plots. Statistical significance was determined by two-tailed Student’s t-test (****p* < 0.001). For panels A, C, and D, only significantly different functions are shown, with significance calculated using DESeq2. For panel E, data across different time points were analyzed using one-way ANOVA followed by Tukey’s post hoc test; different superscript letters indicate significant differences.

**Figure S3 ^15^N-urea isotope tracing reveals reduced host assimilation of microbiota-derived nitrogen following LPD-derived virome transplantation.**
(A) Schematic of the experimental design for tracing nitrogen flux. Recipient mice received FVT from LPD donors. After a 2-week adaptation period to allow microbiome metabolic restructuring, mice were administered ^15^N-labeled urea in the drinking water for 1 week. Liver tissues were harvested, acid-hydrolyzed, and analyzed by LC–MS to quantify incorporation of microbiota-derived ^15^N into host amino acids.
(B) ^15^N-labelled amino acid content in mouse liver. Upper panel shows essential amino acids; lower panel shows non-essential amino acids (n = 5).

(C) Quantification of faecal bacterial abundance in the ND and FL groups.

(D) Growth of ND and FL group bacteria in urease screening medium, measured by OD600 at different time points (left, compared with the ND group, bacteria from the FL group exhibited significantly lower growth at all time points from 6 h to 36 h), and free ammonia concentrations in culture supernatants after incubation (right).

For panel B and C, statistical significance was assessed using two-tailed Student’s t-test or Welch’s t-test where appropriate; non-parametric comparisons were performed using the Mann–Whitney U test (**p* < 0.05, ****p* < 0.001, *****p* < 0.0001).

**Figure S4 ND-derived virome transplantation does not restore LPD-associated bacterial metabolic functions.**
(A) Differential abundance of genes involved in carbohydrate metabolism. Compared with the LPD microbiome, FN treatment is associated with a reduced abundance of genes related to carbohydrate degradation (n = 4).
(B) Differential abundance of genes related to protein and amino acid metabolism. FN treatment does not enhance bacterial protein metabolic capacity (n = 4).
(C) Gene set enrichment analysis (GSEA) showing significant enrichment of the “Amino sugar and nucleotide sugar metabolism” pathway in the FN group (enrichment score = 0.6167), a pathway involved in the biosynthesis of nucleotide sugars and peptidoglycan precursors (n = 4).

Differential abundance analyses in panels A and B were performed using DESeq2, with only significantly different functions shown.

**Figure S5 Protein availability dictates phage release kinetics and lytic activity.**
(A) Phage release kinetics of fecal bacteria cultured in nutrient-rich GAM-BB medium. Representative flow cytometry plots.

(B) Quantified virus like particle (VLP) counts show that LPD-derived bacteria exhibit a delayed but rapid surge in phage release after 60 min, surpassing ND-derived bacteria (n = 3).
(C) Quantification of spontaneous phage release from equalized bacterial numbers (10⁶ cells). Baseline VLP counts during the exponential phase are comparable between ND and LPD groups (n = 3).

(D) Effect of nutritional conditions on phage release. Phage release was measured under normal (Peptone 12 g/L) and low-protein (Peptone 3 g/L) conditions. ND-derived bacteria show significantly reduced phage release under protein-limited conditions (n = 3).

Statistical significance in panels B and D at each time point was determined using a two-tailed Student’s *t*-test (***p* < 0.01, ****p* < 0.001).
